# Better than nothing? maternal, newborn, and child health services and perinatal mortality, Lubumbashi, democratic republic of the Congo: a cohort study

**DOI:** 10.1186/s12884-016-0879-y

**Published:** 2016-04-26

**Authors:** Abel Mukengeshayi Ntambue, Françoise Kaj Malonga, Michele Dramaix-Wilmet, Roger Nlandu Ngatu, Philippe Donnen

**Affiliations:** 1 Université de Lubumbashi: École de Santé Publique, Unité d’Epidémiologie et de Santé de la Mère, du Nouveau-né et de l’enfant, Lubumbashi (DRC), Democratic Republic of the Congo; 2 Université Libre de Bruxelles: École de Santé Publique: Centre de recherche en Epidémiologie, Biostatistiques et recherche clinique, Brussels, Belgium; 3 Graduate School of Health and Nursing Sciences & Disaster Nursing Global Leader doctoral program (DNGL), University of Kochi, Kochi, Japan; 4 Université Libre de Bruxelles: École de Santé Publique: Centre de Recherche en Politiques et systèmes de santé-Santé internationale, Brussels, Belgium

**Keywords:** Perinatal mortality, Maternal health services, Perinatal care, Postnatal care, Emergency obstetric and neonatal care, Democratic Republic of the Congo

## Abstract

**Background:**

The Democratic Republic of Congo (DRC) has a high rate of perinatal mortality (PMR), and health measures that could reduce this high rate of mortality are not accessible to all women. Where they are in place, their quality is not optimal. This study was initiated to assess the relationship between these suboptimal maternal, newborn and child health (MNCH) services and perinatal mortality (PM) in Lubumbashi, DRC’s second-largest city.

**Methods:**

We conducted a prospective cohort study, comparing women who had no, low, moderate, or high numbers of antenatal care (ANC) visits; three different levels of delivery care; and who did or did not attend postnatal care (PNC). Women were followed for 50 days after delivery, with PM as the primary endpoint.

**Results:**

Uptake of recommended prenatal interventions was between 11-43 % among ANC attenders, regardless of the frequency of their visits. PM was 26 per 1000. ANC attendance was associated with PM. Newborns of mothers who had the lowest attendance had a mortality two times higher than newborns of women who had not attended ANC (low visits: adjusted odds ratio (aOR) = 2.2; 95 % confidence interval (CI) = 1.4-3.8). However, moderate (aOR = 1.4; 95 % CI =0.7–2.2) and high (aOR = 1.3; 95 % CI 0.7–2.2) attendance were not statistically significantly associated with PM. PNC attendance was not significantly associated with lower PM (relative risk 0.4, 95 % CI 0.1–2.6). Emergency obstetric and newborn care (EmONC) was significantly associated with a reduction in mortality (aOR = 0.2; 95 % CI = 0.2–0.8), with an 84.4 % reduction among newborns at risk, and an overall reduction in mortality of 10 % for all births.

**Conclusion:**

Perinatal mortality was high among the infants of women in the cohort under study (26 per 1000 live births). Availability of MNCH, specifically EmONC, was associated with lower perinatal mortality, and if this association is causal, might avert 84.4 % of perinatal deaths among newborns at high-risk.

## Background

The Democratic Republic of Congo (DRC) has one of the highest perinatal mortality rates (PMR) in the world (>40 per 1000) [1]; maternal, neonatal, and child health (MNCH) services are rarely used and are of poor quality [2, 3]. As in other countries [4], there are differences between rural and urban areas [3]. Lubumbashi, the second largest city, has a PMR greater than 27 per 1000, with mediocre MNCH utilization [5]. Complicated delivery and low birth weight are the main risk factors for perinatal death [5, 6].

MNCH includes antenatal care (ANC), skilled attendance at delivery and postnatal consultations (PNC), and integrated management of newborn and childhood illnesses (IMCI) [4]. Although the individual impact of certain interventions in the MNCH package remains to be shown, as a whole, this package is an effective intervention to reduce maternal and perinatal mortality (PM) [7–10]. Thus, MNCH care is most effective when the interventions are offered, not as isolated interventions, but in the form of a package on the continuum: pregnancy, delivery, and postpartum [11–13].

In 2008, a meta-analysis by Bhutta et al. [11] showed that quality ANC and appropriate care at delivery and during the neonatal period could reduce maternal mortality by more than 65 %, and PM by 80 %. Here, as elsewhere [4, 7–10, 13, 14], the authors mentioned that the impact of MNCH interventions could only be attained if the care ─ quality ANC with skilled attendance at delivery and postnatal care (PNC)─ was offered as a ‘package’ and on a continuum rather than through isolated interventions. In South Africa, Pattinson et al. [15] observed similar results when women had received appropriate care at each critical phase.

In several settings in Africa, it is still rare for all the components of this package to be offered to all women and newborns throughout the continuum [4]. In Lubumbashi in 2009, while over 90 % of pregnant women used ANC at least once during their pregnancies, less than 30 % received the recommended and/or necessary interventions during these visits (iron, folic acid, sulphadoxine-pyrimethamine, tetanus toxoid, and HIV, syphilis, and intrauterine growth restriction screening) [2]. Although almost all women (97.2 %) had an assisted delivery, the utilization and quality of emergency obstetric and newborn care (EmONC) remained inadequate. Only 7.6 % of births in 2010 took place in health facilities that offered Comprehensive EmONC. Moreover, only 35 % of women had attended PNC at 42 days, and PNC services were restricted to simply weighing and vaccinating the child [2, 16].

These data illustrate the insufficient quantity and quality of MNCH care in this environment, and are consistent with the high PMR observed, as well as with the maternal mortality due to direct obstetric causes and intrapartum and very early neonatal death – indicators of EmONC – which, instead of being below 1.5 %, have generally been higher, above 3 %. They beg the question of what, if any, impact on PM, MNCH care may have in this precarious context. This study was conducted to assess the relation between the MNCH package as currently provided with PM in Lubumbashi.

## Methods

### Study setting

Lubumbashi is the capital of the province of Katanga. In 2010, its population was estimated at 1,548,923 inhabitants in an area of 747 square kilometers [17]. It has 11 health zones (HZ) and 267 health facilities, of which only 180 have a maternity ward [18]. Each HZ has a hospital and comprises both urban and rural areas. Within each HZ, facilities were purposively chosen for inclusion in the study. Each of the ten general reference hospitals (GRH) and the one provincial reference hospital was chosen, and in the corresponding HZ, one health center or private clinic (≥30 deliveries per month) in an urban setting and one in a rural (or urban-rural) setting was selected. Thus, apart from Lubumbashi (1 GRH, 1 provincial hospital, two health centers) and Kowe HZ (one health center), each HZ had 3 facilities participating in the study.

### Study population

#### Selection of subjects

We conducted a prospective cohort study, comparing women who delivered in one of the study facilities and had received various levels of ANC with women who delivered in those same facilities but had not received ANC. The study took place over a 5-month period from October 2010 to February 2011.

Women who did not use ANC were recruited at their admission to the maternity unit. Those who attended ANC were recruited at their first prenatal visit. They were followed at ANC visits. At the end of scheduled prenatal visits, they were sought in the maternity wards where they had planned to give birth according to their expected date of delivery. Women were also encouraged to contact the research team in case of premature birth. Women who had not attended the ANC were recruited at the time of their admission during the same period and in the same maternity units as women of the ANC group.

At the ANC, like in the maternity unit, women were recruited by nurses and doctors trained in the study procedures. The admission of a woman to a maternity unit did not automatically guarantee her inclusion in the study; written informed consent was first obtained.

All women included in the study were followed until 50 days after delivery. The follow-up consisted of verification of care received during the stay in the maternity unit, and, after the stay, use of postnatal care and assessment of maternal and neonatal survival.

In the ANC group, women were monitored to assess the quality of care received during pregnancy. They were recruited during ANC; only pregnant women attending their first visit were enrolled in the study. In Lubumbashi, the minimum number of women expected at ANC annually is about 60,000. Allowing for a significance level of 5 % and loss to follow-up of 20 %, with a power of 80 % to detect a reduction of PM with ANC attendance of ≈ 22.1 % [5], we needed to recruit at least 1812 pregnant women (906 per group). However, taking into account the analysis in subgroups planned to assess the impact of ANC on the maternal-fetal prognosis, the number of women was multiplied by 3 (number of ANC subgroups), which gave a minimum number of 2745 pregnant women to be recruited [19]. The number of women enrolled by each health facility was proportional to the ANC capacity of the facility (in 2009), relative to the total number of women attending ANC during the same year, across all selected health facilities [19, 20].

These women were followed by the research team according to the WHO ANC program [21], operating in the health facilities. During this monitoring period, meetings were held with women in the health facilities on the occasion of their prenatal visits. Women were not sought at home when they were absent from the appointment given by the health personnel. There was no interference from investigators in the organization of activities at a prenatal visit. No intervention was proposed to the women during this period. The fiftieth day post-partum marked the end of this monitoring period.

During the monitoring period, the investigators interviewed women to obtain information about the date of their last menstrual periods (LMP), their use of home health practices, and advice and examinations they received during ANC visits. Investigators also reviewed ANC records to record which recommended interventions were administered, obstetric history, and morbidity during pregnancy.

A total of 2823 pregnant women was recruited into this group. During the follow-up period, 5 women (0.2 %) experienced a miscarriage, and 424 (15 %) were lost to follow-up (not found even at home). There was no statistically significant difference in the profile of the women who remained in the study and those lost to follow-up. No maternal deaths were recorded before delivery. At delivery, the remaining 2,394 women were compared with those who had not attended ANC.

According to the model of focused ANC, women should make at least four prenatal visits [21, 22]. The interventions received depend not only on the frequency of these visits, but on when the visits occur. For most developing countries, the recommended gestational ages for ANC attendance are ≤ 16 weeks, ≤ 28 weeks, ≤32 weeks, and ≤ 36 weeks since LMP [21, 22].

Thus, if a woman gives birth prematurely, at 34 weeks, she would be expected to have made three visits to ANC. We calculated the adequacy of visits according to gestational age at delivery to classify women as low, moderate, or high ANC attenders. A low ANC attender was one who attended only one of either 3 or 4 expected visits, for an adequacy of 25 or 33 % (912 women, 38.1 %). A moderate ANC attender was one who attended one of two or three of 3 or 4 expected visits, for an adequacy of 50, 66, or 75 % (748 women, 31.2 %). A high ANC attender was one who attended all, or more than, the expected visits (734 women, 30.7 %). There were 1,910 women who delivered at a study facility but who had not attended ANC, for a total of 4,304 women in the study.

Written informed consent was obtained from each woman prior to inclusion in the study and was reassessed at each contact. Literacy is low in DRC (a recent estimate put it at 67 %), especially among women (52 %), and especially among those with less education and lower socioeconomic status [3]. For those whose reading ability and understanding of French were low, the consent form was read and explained to the woman in one of the local languages (Swahili, Tshiluba or Lingala), in the presence of a witness. This protocol was approved by the Comité d’Éthique Medicale (CEM; Medical Ethics Committee) of the University of Lubumbashi (CEM-UNILU: UNILU/CEM/010/2011).

#### Monitoring of women and data collection

The women were followed from their admission to the maternity unit until 50 days after delivery. Women in the ANC group were sought in maternity facilities where they had followed the ANC program at the time of their estimated date of delivery. Those with a telephone were contacted at this date to confirm their stay in the maternity facility. Women not found in the facility where they had attended ANC (11.8 %) were sought at their homes.

At the maternity facility, we interviewed women to get data relating to pregnancy for women who had not attended ANC, and the circumstances in which the woman had arrived at the facility, including time of leaving home, means of transport, and difficulties at admission for both groups of women. From maternity records, we collected data related to the diagnosis at admission, type of pregnancy, fetal presentation, mode of delivery, the occurrence of complications, the weight of the newborn and the fetal-maternal prognosis. During an interview with the health staff who managed the delivery, a table with information on the organization and access to obstetric and neonatal care in the health facility, as well as the availability of care at the time of delivery, was filled in. This table evaluated the availability of staff with surgical skills, as well as the availability of drugs and equipment (blood, oxytocin, antibiotics, surgical kits, oxygen, an incubator, magnesium sulfate, phototherapy, nasogastric tube, a partograph, an ‘Ambu’ bag, a bulb syringe and vacuum extraction cup) for each delivery. The availability of these latter varied according to the ability of the woman or her family to pay for (≥ US $20–60) and obtain them, and were not characteristics of the facility.

After women and their infants returned home, the study team visited them 8, 30, and 50 days after delivery. At these visits, we assessed the vital status of mother and child and the use of postnatal care at 7, 21 and 42 days after delivery.

#### Data management and definition of variables

The study data were double-entered and analyzed using Stata v.11.0 (College Station, TX). As described above, the use of ANC was evaluated in terms of the number of visits. A woman was considered at high risk of PM if she had at least one complication occurring before, during, or after delivery. Also, a fetus or a newborn was considered to be at high risk of dying if born to a woman at risk, or if it experienced a complication at birth or during the neonatal period [21, 23–25]. To study the impact of the management of delivery on maternal-fetal and neonatal survival, we identified three care groups. The first we called Essential Care–Low Risk (EC-LR), which was the general care given to women and newborns at low risk. This care included clean birth practices, labor monitoring using a partograph, the active management of the third stage of labor (AMTSL), hygienic methods to cut and tie off the cord, basic thermal care, immediate breastfeeding, prevention of mother-to-child transmission of HIV (PMTCT), and increasing the satisfaction and comfort of the mother. The second group received the same care, but were determined to be high-risk; this group was called Essential Care-High Risk (EC-HR). The third group was that which received emergency obstetric and neonatal care (EmONC), as defined by WHO [23, 24]. The PNC visits were evaluated according to whether the women had attended them or not [4].

The level of risk of the pregnancy was determined by the presence during pregnancy of at least one factor of poor prognosis for mother and child. The criteria were those of the WHO, as well as others observed by us previously in Lubumbashi [5, 23]. They included age < 20 and > 37 years, primigravidity, miscarriage of the previous pregnancy, previous infant born at less than 2500 g, fetal or neonatal death at previous delivery, the occurrence of complications or Caesarean section at previous delivery, hypertension, diabetes, maternal malnutrition, sexually transmitted infections, or HIV during the current pregnancy, and type of pregnancy (singleton vs. multiple pregnancy). Thus, every woman having least one of these factors or complications was considered as having a high-risk pregnancy [23].

PM was defined as all stillbirths (≥28 weeks gestational age) and early neonatal deaths (≤7 days) [26]. For women who had multiple pregnancies, only one infant was selected at random to be included in the univariate and multivariate analyses.

### Data analysis

Usual descriptive statistics were used to describe groups. Comparisons of the mean or median between groups involved ANOVA or Kruskal-Wallis tests, as appropriate [20]. The association between use of MNCH services and PM was measured by calculating a relative risk (RR), the significance of which was tested using a chi-square test, with a significance level of 5 %. The adjusted odds ratios (aOR) were calculated by forward stepwise logistic regression. Confidence intervals at 95 % were calculated for crude RR and aOR. All variables with an unadjusted *p*-value ≤ 0.10 had the opportunity to enter into the multivariable logistic regression analysis. Model fit was checked by the Hosmer-Lemeshow test [27].

The impact of each MNCH component in reducing PM was assessed by the fraction of risk prevented in newborns at high risk (FRP_c_) and the fraction of risk prevented in all births (FRP_A_) for all variables significantly associated with the reduction of PM. The total fraction of risk prevented, FRP_t_, for all MNCH was calculated using the following formula:

$$ \mathrm{F}\mathrm{R}{\mathrm{P}}_{\mathrm{t}} = 1\hbox{-} \left[\Big(1\hbox{-} \mathrm{F}\mathrm{R}{\mathrm{P}}_{\mathrm{S}1}\right)\left(1\hbox{-} \mathrm{F}\mathrm{R}{\mathrm{P}}_{\mathrm{S}2}\right)\left(1\hbox{-} \mathrm{F}\mathrm{R}{\mathrm{P}}_{\mathrm{S}3}\right)\ \left(1\hbox{-} \mathrm{F}\mathrm{R}{\mathrm{P}}_{\mathrm{S}4}\right)\Big] $$

Where FRP_Si_ = fraction of risk prevented with a given MNCH service [28–31].

## Results

### Characteristics of subjects

**Fig. 1:**
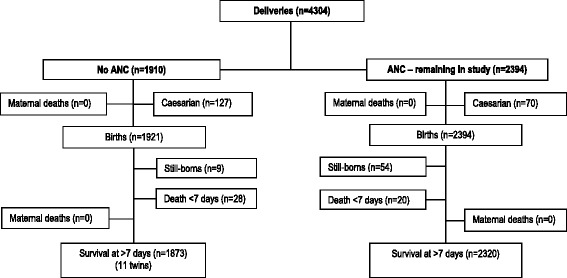
Numbers of women and newborns according to their prognosis at childbirth and during the neonatal period

At delivery, 4,304 women were included in this study (Fig. 1). Of these, 2,394 (55.6 %) were in the group followed during ANC, while 1,910 others (44.4 %) had not received ANC and were recruited in the delivery room. No maternal deaths occurred during or within 42 days of childbirth.

A total of 4,315 children were born to these women; 1,921 (44.5 %, including 22 twins from 11 multiple pregnancies), were born to mothers who had not attended the ANC program, and 2,394 (55.5 %) were born to mothers who had attended.

The total number of fetal deaths in this cohort was 63 (14.6 per 1000 births), 9 in women who had not attended the ANC program (0.47 %), and 54 in those who had attended (2.3 %). A total of 48 early neonatal deaths occurred; 28 of these deaths (58.3 %) occurred among infants born to women who did not attend the ANC (28/1910 = 1.5 %; 20/2394 = 0.84 %). The PMR was 25.3 per 1000 births. At the end of the first week of neonatal life, the total number of surviving infants was 4,204. This number consisted of 1,884 (1884/1921 = 98.1 %) neonates born to mothers who had not attended the ANC program and 2,320 (2320/2394 = 96.9 %) to mothers who had attended (Fig. 1).

The mean age of women in both groups was 26 years (standard deviation (SD) 6). Women who had not attended ANC were on average a year or two younger than those who had (no ANC, 25.1 (SD 6.7) years vs low visits, 26.9 (SD 6.2), moderate visits, 26.4 (SD 5.9), and high visits 26.4 (SD 5.9) years). In Table 1, it can be seen that there were more unmarried women among those who had not attended ANC than those who had. Similarly, the proportion of women working at home was higher in the group with high attendance than in women who did not attend ANC.

**Table 1:** Socio-demographic and obstetric characteristics of women by level of antenatal care (ANC) attendance, Lubumbashi, DRC, 2010–11

Characteristics	No ANC (*n* = 1910) %	ANC Visits			
		Low (*n* = 912) %	Moderate (*n* = 748 %	High (*n* = 734) %	*p*
Age (Years)					<0.001
<20	23.6	11.5	11.0	10.9	
20–34	64.7	73.9	77.2	77.1	
≥35	11.7	14.6	11.8	12.0	
Marital status					<0.001
Married	95.2	97.7	97.5	98.5	
Unmarried	4.8	2.3	2.5	1.5	
Education					<0.001
None or primary	27.6	25.9	24.7	21.8	
Secondary school	69.5	68.5	69.4	69.6	
University	2.9	5.6	5.9	8.6	
Occupation					0.032
Housework	54.9	59.4	55.9	60.1	
Other	45.1	40.6	44.1	39.9	
High-risk pregnancy	43.3	43.3	32.5	42.5	<0.001
<18 yrs	17.5	8.3	7.1	7.0	<0.001
>37 yrs	7.4	8.1	6.8	6.1	<0.001
≥6 previous deliveries	12.8	20.3	16.8	17.0	<0.001
Poor OH^a^	10.6	12.1	9.6	15.3	<0.001
Multiple pregnancy	0.6	0.0	0.0	0.0	

*OH* obstetrical history

^a^: poor obstetrical history (low birth weight, stillborn, abortion)

Women who had high attendance were more highly educated than those who did not receive ANC. The proportion of women with high-risk pregnancies was lowest in the moderate-attendance group. Among the factors associated with high-risk pregnancies were early motherhood (<18 years), high parity, and poor obstetric history in the preceding pregnancy and delivery. Early motherhood was twice as high in the group without ANC as in the one with ANC. In contrast, the high parity and poor outcomes of previous pregnancy were more frequent in the group with ANC than in the one without ANC. Only 11 of the 4,304 women enrolled had a twin pregnancy, all of whom were in the group with no ANC attendance.

During pregnancy, it was the women who had high ANC attendance who received more recommended prenatal health interventions. However, even among women at the highest level of ANC attendance, interventions such as HIV screening and tetanus vaccination were well below 50 %. Overall, nearly 60 % of all women were recruited in the general referral hospitals. The place of delivery was not significantly associated with the adequacy of prenatal visits (Table 2). In Table 3, it can be seen that there were more complications during delivery in the group without ANC than with ANC. Similarly, there were more fetuses or newborns that developed complications at birth or in the neonatal period among women who had not attended ANC than among those who had. The nature of the complications varied according to the adequacy of visits. Among women who had not attended ANC, post-partum hemorrhage was the most common complication observed, followed by prolonged labor (4.3 %) and eclampsia (1.8 %). Prolonged labor and eclampsia were the most often observed complications among women who had low or moderate ANC attendance. Besides the complications commonly seen in women who had low attendance, uterine rupture was frequently seen in women who had high attendance. There was no post-partum hemorrhage in women who had received ANC.

**Table 2:** Properties of the ANC program and the place of delivery by level of antenatal care (ANC) attendance, Lubumbashi, DRC, 2010–11

Characteristics	No ANC (*n* = 1910) %	ANC Visits			
		Low (*n* = 912) %	Moderate (*n* = 748) %	High (*n* = 734) %	*p*
Health interventions during ANC					
Sulfadoxine- Pyrimethamine	0.0	22.5	35.3	37.6	<0.001
Iron	0.0	24.8	31.0	29.0	0.015
Folic acid	0.0	17.9	21.1	22.1	0.08
ITN	0.0	19.5	23.8	17.9	0.013
Mebendazole	0.0	29.7	29.0	27.7	0,65
Antitetanus Vaccination	0.0	35.1	43.1	41.8	0.002
HIV screening	0.0	11.8	13.6	11.2	0.32
Place of delivery					0.39
Health center	42.6	45.3	42.2	41.4	
General Referral Hospital	57.4	54.7	57.8	58.6	

*ITN* Insecticide-treated net

**Table 3:** Adequacy of prenatal care based on maternal and neonatal complications and use of obstetric, neonatal and postnatal care by level of antenatal care (ANC) attendance, Lubumbashi, DRC, 2010–11

Characteristics	No ANC (*n* = 1910) %	ANC Visits			
		Low (*n* = 912) %	Moderate (*n* = 748 %	High (*n* = 734) %	*p*
Maternal complications	15.2	5.9	6.6	11.6	<0.001
Fever	2.1	0.5	0.8	0.8	<0.001
Premature rupture of membranes	0.6	0.8	1.1	1.1	<0.001
Severe anemia	0.4	0.6	1.1	1.1	<0.001
Dystocia (prolonged labor)	4.3	1.2	1.6	3.3	<0.001
Eclampsia	1.8	1.1	0.8	0.5	<0.001
Placenta abruptio	0.1	0.1	0.1	0.3	<0.001
Placenta Prævia	0.0	1.0	0.6	2.5	<0.001
Uterine rupture	0.2	0.6	0.5	2.0	<0.001
Postpartum hemorrhage	5.4	0.0	0.0	0.0	^a^
Neonatal Complications	26.5	16.9	13.9	20.6	<0.001
Respiratory distress (Apgar < 7)	17.2	11.9	11.1	15.1	
Low birth weight	9.3	5.0	2.8	5.5	
Obstetrical care					<0.001
EC-LR	84.8	94.1	93.5	88.4	
EmOC	9.9	2.7	2.1	6.3	
EC-HR	5.6	3.2	4.4	5.3	
Neonatal Care					<0.001
EC-LR	73.5	88.1	86.1	79.4	
EmNC	15.2	6.7	4.9	10.5	
EC-HR	11.3	10.2	9.0	10.1	
Postnatal consultation^b^	6.9	3.4	5.2	5.9	0.003

^a^not calculated; ^b^ *n* = 4241

Maternal and neonatal complications were used to determine the proportion of fetuses and/or newborns at high risk. Considering complications occurring in the mother and those occurring in newborns together, the proportion of fetuses or newborns at risk was 21.3 % (917 of 4304). It was higher in the group without ANC than for those with any ANC. Low birth weight (LBW), defined as < 2500 g, and respiratory distress syndrome (RDS) were frequently observed complications. The proportion of newborns with LBW was nearly two times higher in the group without ANC than in the one with any ANC. There was no statistically significant difference in the proportion of newborns with RDS among newborns of women who had attended ANC and women who had not.

Regarding the management of care, the proportion of women who received emergency obstetric care (EmOC) was higher in the group without ANC than in the one with ANC. Similarly, newborns of mothers who had not received ANC received more emergency neonatal care (EmNC) than those of mothers who had received ANC (all categories). The proportion of women who had used PNC within 7 days was higher among women who had not followed the ANC than among those with ANC.

### Relation between MNCH and fetal and neonatal prognosis

From the unadjusted analysis in Table 4, it appears that women who had occupations other than housework had 1.5 times the PMR as those who were only occupied in the home. The PMR was twice as high among infants of mothers who had previously experienced a high-risk pregnancy compared with those whose mothers had no risk in previous pregnancies, and newborns of mothers who experienced birth complications also had 4 times the PMR as mothers who did not. Newborns who had complications at birth or during the first week of life were at 5.4 times higher PMR than those who had not.

**Table 4:** Perinatal mortality based on maternal characteristics and maternal and neonatal complications: unadjusted analysis, by level of antenatal care (ANC) attendance, Lubumbashi, DRC, 2010–11

Factors	Total	Rate (per 1000)	RR	95 % IC	*p*
Marital status					0.85
Married	4162	26.0	1		
Unmarried	142	28.2	1.1	0.4–2.9	
Education					0.07
None or primary	1108	31.6	1.3	0.8–1.9	
Secondary school	2983	25.1	1		
University	213	5.0	0.2	0.1–1.3	
Occupation					0.030
Housework	2450	21.2	1		
Other^a^	1854	32.0	1.5	1.1–2.2	
Status of pregnancy					<0.001
High risk	1778	36.6	2.0	1.4–2.9	
Low risk	2526	18.2	1		
Maternal complications					<0.001
Yes	478	77.4	4.0	2.7–5.9	
No	3826	19.3	1		
Neonatal Complications					<0.001
Yes	916	72.1	5.4	3.7–7.9	
No	3388	13.3	1		

*RR* Relative Risk

^a^civil servant, agriculture, sales, works in business or industry

In Table 5, we note that women who had low attendance had twice the PMR as those who did not attend ANC. The PMR was reduced by two-thirds among infants at high risk whose mothers had received EmOC compared to infants at high risk whose mothers had received only EC-HR. Similarly, newborns at high risk who received EmNC were more likely to survive than those at high risk who received standard care. Although the difference was not significant, the PMR was lower among infants whose mothers had attended the PNC compared to those whose mothers had not attended this service. The type of facility in which delivery occurred was not associated with mortality.

**Table 5:** Perinatal mortality in terms of the services available to the mother, to the newborn and to the child: unadjusted analyses

Factors	Total	Rate (per 1000)	RR	95 % CI	*p*
Type of maternity facility					0.39
Health center	1846	28.2	1.2	0.8–1.7	
General Referral Hospital	2458	24.0	1		
ANC (adequacy of visits)					0.006
None	1910	19.4	1		
Low	912	41.7	2.1	1.4–3.4	
Moderate	748	24.1	1.2	0.7–2.2	
High	734	24.5	1.3	0.7–2.2	
Obstetrical care					<0.001
EC-LR	3826	19.3	0.1	0.08–0.2	
EmOC	270	44.4	0.3	0.2–0.7	
EC-HR	208	120.2	1		
Neonatal Care					<0.001
EC-LR	3388	13.3	0.1	0.08–0.2	
EmNC	466	42.9	0.4	0.2–0.8	
EC-HR	450	102.2	1		
PNC ≤ 7 days^a^					0.27
Yes	243	4.1	0.4	0.1–2.6	
No	3998	11.8	1		

*RR* Relative Risk

^a^ *n* = 4241

From the multivariate analysis (Table 6), it can be seen that women who had low ANC attendance had a higher PMR than those who had not attended ANC, while the risk did not differ significantly for those who were moderate or high attenders. EmOC for women who had complications reduced PMR by more than half. There was 62.3 % less PMR for such women than for those who had not. Similarly, newborns at high risk who received EmNC had 58.7 % less PMR than those who did not receive such care.

**Table 6:** Perinatal mortality according to MNCH: adjusted analyzes and risk in infants at high risk and among all births

Factors	aOR	95 % CI	*p*	Surviving % (*n* = 4193)	FRP_c_	FRP_A_
Education			0.07			
None or primary vs Secondary school	0.8	0.5–1.2				
University vs Secondary school	0.2	0.1–1.1				
ANC (adequacy of visits)			0.010			
Low vs No ANC	2.2	1.4–3.8				
Moderate vs No ANC	1.4	0.7–2.2				
High vs No ANC	1.3	0.7–2.2				
Presence of partograph (Yes vs No)	1.1	0.7–1.7	0.58			
General Reference Hospital vs. Health Center	0.8	0.5–1.2	0.23			
High-risk pregnancy vs low risk	1.9	1.3–2.9	0.002			
Obstetrical care			<0.001			
EC-LR vs EC-HR	0.3	0.2–0.5		89.5	71.3	63.8
EmOC vs EC-HR	0.4	0.2–0.8		6.1	62.3	3.8
Neonatal care			<0.001			
EC-LR vs EC-HR	0.2	0.1–0.3		79.7	83.3	66.4
EmNC vs EC-HR	0.4	0.2–0.8		10.6	58.7	6.2
EmONC					84.4	10.0

By calculating the fraction of risk prevented in infants at high risk, it appears that 84.4 % of perinatal deaths were avoided by EmONC, which accounts for 10 % of deaths avoided among all births.

## Discussion

In our results, we observed an elevated PMR among newborns of women who attended ANC compared to those of women who did not. We noted, however, that the newborns who had received emergency neonatal care and those of women who had received EmOC had a lower PMR than those of women who had not received EmOC. These results corroborate the estimates provided by Darmstadt et al. [12] regarding the prevention of PM from the model for MNCH continuity. They are also in line with the observations of Bhutta [11] and Pattinson [15] who showed that MNCH services, when continuously available, reduce PM by more than 35 % in the general population, or by 85 % among infants at high risk. Nonetheless, it remains debatable whether we can evoke a causal role for MNCH care in the reduction of PM.

In fact, even with intervention studies, evidence is insufficient concerning the individual impact – in terms of a causal role – of each component of prenatal, perinatal, and postnatal care on the reduction of PM, even if globally this reduction is observed among the women and newborns who receive a high-quality package of MNCH care [7–12, 32]. Such is the case for our study, in which the low PMR was observed among the newborns who had received adequate care. The package of neonatal and maternal care was put in place based on the convergence of expert opinion about the averred or presumed effectiveness of the interventions and their synergy, although solid scientific evidence, in terms of the specific contribution of each one, is yet to be found [32–37].

### During pregnancy

The impact of interventions offered through ANC (screening for high-risk pregnancy, micronutrient supplementation, intermittent presumptive treatment (IPT) and long-lasting insecticide treated bed nets (LLIN), management of chronic hypertension, screening and treatment of syphilis) on the reduction of PM has not been proven for some and remains to be qualified for others [32–44].

On the other hand, screening for syphilis, IPT and LLIN, screening and treatment of diabetes over the course of pregnancy, prenatal corticoids as well as screening and induction of labor in cases of intrauterine growth retardation (IUGR) are, of all these interventions, the ones for which there is some proof in terms of impact on reduction of PM [45–52].

As to screening and management of diabetes mellitus, some prospective studies have shown that adequate glycemic control and follow-up during pregnancy are a reasonable means to reduce stillbirth and numerous other complications like congenital anomalies and macrosomy [51–53]. The effect of prenatal corticosteroids on fetal maturity has been extensively documented and it has been observed that this intervention reduced respiratory distress syndrome (RDS) by 34 %, cerebro-ventricular hemorrhage by 46 %, and neonatal death by 31 % [54]. Induction of labor for pregnancies past 41 weeks of gestation has been associated with a 69 % reduction in PM as well as the aspiration of meconium [55, 56].

In the DRC and in the province of Katanga, the prevalence of syphilis among pregnant women is 1.9 and 0.8 %, respectively [57]. This infection is not yet systematically detected during pregnancy [2], which means that whether women have attended ANC or not, the risk of fetal death from the infection remains unchanged because ANC services have not been financed to test for it. This is also the case for malaria, which affects more than 30 % of pregnant women and for which only 30 % receive IPT [5], gestational diabetes and IUGR, which are still rarely screened during pregnancy in Lubumbashi. Since this factor is significantly associated with PM [16], and as long as women who have them fail to receive proper care, it is clear that ANC cannot make a difference to the level of PM between those who attend and those who do not.

However, apart from the particular interventions highlighted above, the impact of ANC on PM appears indirect. Women who attend ANC are more likely to give birth in the presence of skilled health personnel than those who do not [58]. Given that delivery complications occurred as often in women who attended ANC as in those who did not, ANC alone is not likely to reduce this mortality. Thus, if the woman attended ANC and gives birth in the presence of skilled health personnel, she is more likely to receive appropriate care during delivery and postpartum, which helps to improve her prognosis and that of her newborn.

### Delivery and the postnatal period

In Lubumbashi, more than 70 % of perinatal deaths occurred between birth and the 7th postnatal day, whereas for stillbirths, half of the deaths were during delivery [5]. This implies that the interventions most likely to significantly reduce PM are those given during the delivery and up to the 7^th^ post-natal day rather than before delivery [9, 32–37, 59–62]. This is especially true as, during this period, these deaths are usually due to complications of delivery, including prolonged labor, eclampsia, *placenta abruptio*, *placenta previa*, and umbilical cord prolapse [63], prematurity and low birth weight, and infections [64].

At this critical moment, the impact of care on the reduction of PM, varies from one intervention to the other. For example, the available results on the use or not of the partogram for surveillance of spontaneous labor at term, or different versions of the partogram with different positioning of the line of action on the results of labor have not shown significant differences in terms of the rate of cesareans and the perinatal or maternal prognosis [65]. However, studies evaluating the impact of EmONC abound. Even though intervention studies are rare, observational studies, taken as a whole, indicate that skilled birth attendance with providers capable of safe and timely provision of all the signal functions of EmONC are essential to reduce the PMR [59, 66, 67]. The quality of EmONC, rather than its simple availability, is essential in the prevention of PM. The available data show that emergency cesarean and assisted vaginal delivery by ventouse contribute enormously to the reduction of stillbirth. This interventions are signal functions of EmONC with a high impact in the prevention of PM tied to direct delivery complications [68, 69].

During the neonatal period, interventions include prevention of hypothermia, management of RDS, pneumonia, infection, hyperbilirubinemia, as well as early initiation of breastfeeding [69]. This package offered in a hospital setting to newborns, especially those with LBW, is associated overall with a 51 % reduction in neonatal mortality; 58 % by antibiotic administration, and 77 % by prevention of hypothermia [8]. Neonatal resuscitation in health facilities reduces PM by more than 30 % [32, 70]. Evidence from low-income countries show that prevention and management of hypothermia reduces early neonatal mortality by about 25 % [32, 70–72].

In our study, we observed that a significant proportion of women and newborns with complications receive inadequate care in Lubumbashi. This was due to lack of consumables, medicines and equipment, inadequate training, and lack of protocols for the management of complicated deliveries. Further, many women who arrive late in the reference hospital maternity unit often come from primary care facilities after a significant delay from the onset of complications. To the extent that, even in reference hospital maternity units emergency kits are often lacking (it is the families who have to buy them, at a cost of ≈ US $20–60), it is obvious that not all referred women receive appropriate care. Thus it has happened that, in the same delivery room, of 2 infants with severe respiratory distress, only one survived because oxygen was available, and the other died because the oxygen had run out. Also, since kangaroo mother care is not widely disseminated in health care facilities, the staff tend to keep the very low birth weight (less than1500 g) infants in the incubator, while those between 1500–2500 g are not kept warm well. If the complications of extreme prematurity will kill the very low birth weight infant, it’s often hypothermia that kills those between 1500 and 2500 g.

Regarding PNC, although the difference was not significant, we also found that the PMR tended to be higher in women who had not attended PNC than for those who had attended PNC. This lack of significant association may result from the low power of the tests given the very small number of perinatal deaths among women who followed PNC (Table 5).

All the above observations concerning MNCH care show that it is precarious in Lubumbashi and that the packages are not offered in such a way as to attain their maximum impact. But our observations also call attention to the fact that when the recommended care is given to mothers and newborns, even in this context where organization and work are difficult, it contributes nonetheless to reduction of PM [32, 72]. However, as stated above, it is not easy or possible to determine which interventions in the package of EmONC and care of ill and LBW infants has contributed most to reduce PM in Lubumbashi. It is likely that there exists a synergy of mutual reinforcement among the interventions, while others had an indirect role in the reduction of PM through an effect on factors intimately associated with it. For example, the administration of prenatal corticosteroids is associated with reduced need for ventilation, and reduces the mortality attributable to RDS up to 53 % in settings where oxygen and ventilation is not always available, as in Lubumbashi [70–72]. Synergy between interventions thus reinforces the idea by which, the more the interventions are offered as a package, all along the continuum and at critical points in the life of the mother and child, the more likely they are to be effective and to reduce PM.

### Limitations

This work, which is an observational study, has limitations that are important to present. Although the research team did not interfere in the organization of activities during ANC sessions, or in the use of health interventions by pregnant women, it is unclear to what extent our presence in structures where women were recruited may have been perceived as an audit by the personnel, leading them to change their practices. Likewise, we cannot tell whether the interviews conducted with women at each visit influenced their behavior vis-a-vis the ANC. However, taking into account the organizational constraints of ANC observed in Lubumbashi, such as lack of consumables, drugs and equipment, and also the inaccessibility to pregnant women of certain interventions for financial reasons, despite their availability [2], we believe that the presence of the investigators had no significant influence, in that it did not change either the organization or the financial ability of women to access recommended treatments.

As to the 11 sets of twins born only to women who did not attend ANC, we believe this is a chance finding; we have no particular explanation for it. The same is true of post-partum hemorrhage, which occurred only among women who had not attended ANC. Worldwide, incidence of PPH is 6 %; it is 10 % in Africa [63]. The low incidence of PPH observed in Lubumbashi in this study, compared to that reported in Africa, may be due to differential access to health care. In Lubumbashi, ≈94 % of women have an institutional delivery and ≈ 70 % undergo active management of the third stage of labor. The higher use of this practice may have an effect on the reduction of PPH, which could explain the difference between the incidence reported in our study and that observed at the level of Africa as a whole.

## Conclusion

Perinatal mortality was high among the infants of women in the cohort under study (26 per 1000 live births). MNCH was associated with a reduction in perinatal mortality, specifically through EmONC, which might account for a reduction of 84.4 % in perinatal deaths among newborns at high-risk.

### Ethics approval and consent to participate

Written informed consent was obtained from each woman prior to inclusion in the study and was reassessed at each contact. The study protocol was approved by the Comité d’Éthique Medicale (CEM; Medical Ethics Committee) of the University of Lubumbashi (CEM-UNILU: UNILU/CEM/010/2011).

## Availability of data and materials

My data is available upon request.

## Abbreviations

AMTSL: active management of the third stage of labor
ANC: antenatal care
ANOVA: analysis of variance
aOR: adjusted odds ratios
CEM: Comité d’éthique médicale
CI: confidence interval
DRC: Democratic Republic of the Congo
EC-HR: essential care-high risk
EC-LR: essential care–low risk
EmNC: emergency neonatal care
EmOC: emergency obstetric care
EmONC: emergency obstetric and newborn care
FRP: fraction of risk prevented in newborns with complications
FRP_P_: risk prevented in all births
GRH: general referral hospitals
HC: health center
HIV: human immunodeficiency virus
HZ: health zone
IMCI: integrated management of newborn and childhood illnesses
IPT: intermittent presumptive treatment
IUGR: intrauterine growth retardation
LBW: low birth weight
LLIN: long-lasting insecticide treated bed nets
LMP: last menstrual period
MNCH: Mother Newborn and Child Health
PM: perinatal mortality
PMR: perinatal mortality rate
PMTCT: prevention of mother-to-child transmission (of HIV)
PNC: postnatal consultations
PPH: postpartum hemorrhage
PROM: premature rupture of membranes
RDS: respiratory distress syndrome
RPR: rapid plasma reagin
RR: relative risk
SD: standard deviation
UNILU: Université de Lubumbashi
US $: United States dollars
WHO: World Health Organization
